# Knowledge and Attitude of Primary School Teachers toward Tooth Avulsion and Dental First Aid in Davangere City: A Cross-sectional Survey

**DOI:** 10.5005/jp-journals-10005-1110

**Published:** 2011-04-15

**Authors:** Sapna Prasanna, Anjan Giriraju, Nagesh Lakshmi Narayan

**Affiliations:** 1 Assistant Professor, Department of Preventive and Community Dentistry, Bapuji Dental College and Hospital, Davangere, Karnataka, India; 2 Postgraduate Student, Department of Preventive and Community Dentistry, Bapuji Dental College and Hospital, Davangere, Karnataka, India; 3 Professor and Head, Department of Preventive and Community Dentistry, Bapuji Dental College and Hospital, Davangere, Karnataka, India

**Keywords:** Tooth avulsion, Dental first aid, Primary school teachers, Awareness.

## Abstract

**Aim of the study:**

Traumatic dental injuries including avulsed tooth is a tragic and ignored problem among school children. As children spend much of their time in schools, school teachers form the group who commonly supervise the physical activity of the children, so awareness about avulsed tooth emergency management among school teachers is an important concept for long-term success and to prevent its future consequences. The purpose of this study was to assess the knowledge and attitude regarding tooth avulsion and dental first aid among primary school teachers in Davangere city.

**Methods:**

The study was performed by administering a self-designed questionnaire on a sample of 300 primary school teachers.

**Results:**

Sixty-eight percent of the school teachers (government, semi-aided and aided schools) admitted the possibility of an avulsed tooth to be replanted and thirty-two percent had no idea on tooth replantation and only twenty-three percent of the teachers knew the procedures taken in cases of avulsed teeth. Seventy-seven percent of all teachers did not feel the possibility of tooth replantation.

**Conclusion:**

There is poor knowledge in the management of avulsed teeth among the school teachers of Davangere city. They do not feel capable of replanting an avulsed tooth. As one of the child supervisors, all the school teachers should have the basic knowledge to recognize oral emergencies and regarding conservation of avulsed teeth to prevent its consequences in the child’s future.

## INTRODUCTION

Smile reveals an individual’s self-confidence. It is also a means of socialization where teeth play a vital role in a pleasant smile and also contributes to each individual’s well-being and quality by positively affecting physical and mental well-being, appearance and interpersonal relationship.^1^ Children, especially school children, face particular health challenges including oral health problems related to the stages of their physical and mental development, which makes them especially vulnerable to traumatic dental injuries including the tooth avulsion.

‘Tooth avulsion’ is a situation where, as a result of trauma, a tooth has been removed from the socket. It comprises 0.5 to 16% of all traumatic dental injuries.^2^ In these circumstances, the periodontal ligament fibers and the neuromuscular bundles at the root apex are severed. There may be a concomitant damage to the alveolus and to the tooth. When the tooth is outside the socket, the cells of the pulp and of the periodontal ligament begin to deteriorate. This is due to the effects of a lack of blood supply to the cells and environmental factors (example: drying or bacterial contamination).

Successful replantation of an avulsed tooth depends solely on extraoral drying time and the storage medium of the avulsed tooth.^3^ Clinical outcome studies have demonstrated that the immediate replantation of avulsed tooth is essential for regeneration of periodontal ligament after replantation.^4^

A number of studies have examined the knowledge and attitude regarding tooth avulsion and its immediate management by parents. As the peak age for traumatic dental injuries is between 7 to 12 years and school children are more prone for such injuries, it is essential that all children supervisors like school teachers, school nurses and other school personnel should be well prepared to intervene when such dental emergencies arise.^5^

The purpose of this study was to assess the knowledge and attitude regarding tooth avulsion and dental first aid among primary school teachers of Davangere city.

## MATERIALS AND METHODS

The study was conducted among 300 primary school teachers of Davangere city belonging to government, semi-aided and private schools. They were selected using cluster random sampling method. Permission for the study was obtained from the concerned authorities. The concerned authorities of the selected schools were appraised of the study before taking their permission. The objectives of the study were explained to all the school teachers who participated in the study and also informed consent was obtained from all teachers. A self-designed study proforma containing demographic details, specially framed 10 close-ended questions in English language were personally administered to the teachers. The respondents were then asked to tick the most appropriate answer from the given list of answers, in order to assess their knowledge and attitude regarding the avulsed tooth and dental first aid treatment. Filled questionnaire were collected on the same day. Information regarding the tooth avulsion and its emergency management, as a health talk using power point presentation was given in both English and local language in order to improve the awareness among school teachers. Data collected were analyzed and then were represented in the form of tables.

## RESULTS

The study results showed that when a question was asked about knocked-out tooth 129 (43%) teachers knew what it meant, while 171 (57%) teachers were not aware of it (Table 1).

Regarding the prior knowledge about tooth replantation, 204 (68%) teachers had no knowledge, while 96 (32%) teachers knew what tooth replantation is (Table 2).

When tooth is knocked out and falls on the ground, only 69 (23%) knew what should be done and 231 (77%) teachers did not know what to be done (Table 3).

Regarding the knowledge of teachers about replacement of knocked-out tooth back to its socket was assessed, 72 (24%) teachers answered ‘yes’, 228 (76%) gave ‘no’ as the answer (Table 4).

**Table 1:** **Table 1:** Do you know, what is knocked-out tooth? (Question no.1)

		*Yes*		*No*	
Total		129 (43%)		171 (57%)	

**Table 2:** **Table 2:** Do you know, what tooth replantation is? (Question no.2)

		*Yes*		*No*	
Total		96 (32%)		204 (68%)	

**Table 3:** **Table 3:** If the tooth is knocked out and falls on the ground, do you know what should be done? (Question no.3)

		*Yes*		*No*	
Total		69 (23%)		231 (77%)	

**Table 4:** **Table 4:** Should the knocked out tooth be placed back into the socket? (Question no.4)

		*Yes*		*No*	
Total		72 (24%)		228 (76%)	

**Table 5:** **Table 5:** How immediately the tooth replantation should be performed after the tooth comes out of the socket? (Question no. 5)

*Options*		*Count*		*%*	
5 mins		9		3	
30 mins		33		11	
1 hour		18		6	
6 hours		0		0	
24 hours		3		1	
72 hours		0		0	
I do not know		216		72	
Not answered		21		7	

**Table 6:** **Table 6:** If the tooth falls on the ground and gets dirty, what should you do? (Question no. 6)

*Options*		*Count*		*%*	
Brush its root and crown		18		6	
Wash with tap water		81		27	
Wash it with milk		15		5	
Wash it with saline water		33		11	
Do not wash		18		6	
I do not know		135		45	

Regarding the optimum time within which knocked-out tooth should be replanted, 216 (72%) of teachers answered they were unaware about it, 3 (1%) teachers answered it should be within 24 hours, 18 (6%) as 1 hour, 9 (3%) as within 5 minutes and 33 (11%) of them knew it was within 30 minutes (Table 5).

Regarding the procedure of cleaning the dirty knocked-out tooth, 135 (45%) teachers did not have any idea, 18 (6%) answered you should not wash the tooth, 33 (11%) gave their opinion that it should be washed with saline water, 15 (5%) of teachers answered to wash with milk, 81 (27%) of them suggested on washing it in the tap water, 18 (6%) answered to brush the roots and crown (Table 6).

With respect to the question about the first place of choice for seeking the treatment of the knocked-out tooth, 180 (60%) of them chose to visit the dentist nearby whereas 30 (10%) of them opted emergency hospital for the treatment, 27 (9%) of the teachers would want to consider the emergency management from school of dentistry (Table 7).

Regarding the question on how to transport the avulsed tooth before appropriate treatment, 117 (39%) of the teachers answered cotton rolls as transport media, 81 (27%) did not know about the transportation of the avulsed tooth, 30 (10%) answered tissue paper and saline water, 12 (4%) answered tap water and napkin, 9 (3%) answered as milk, 6 (2%) answered in an envelope and remaining answered keeping the tooth in pocket for transportation (Table 8).

Regarding whether the school teachers had received any information on management of knocked-out tooth, 264 (88%) of teachers had not received any information regarding the management of knocked-out tooth while only a minority 36 (12%) had received such information (Table 9).

**Table 7:** **Table 7:** First place to seek for treatment (Question no.7)

*Options*		*Count*		*%*	
Emergency hospital		30		10	
Local hospital		35		12	
Dentist nearby		180		60	
Private dentist		7		2	
School of dentistry		27		9	
Specialist		4		1	
Others		17		6	

**Table 8:** **Table 8:** Transport media (Question no. 8)

*Options*		*Count*		*%*	
Napkin		12		4	
Tissue paper		30		10	
Cotton rolls		117		39	
Pocket		3		1	
Envelope		6		2	
Tap water		12		4	
Saline water		30		10	
Milk		9		3	
Others		0		0	
I do not know		81		27	

**Table 9:** **Table 9:** Have you ever received any information on management of knocked-out tooth? (Question no 9)

*Yes*			*No*	
36 (12%)			264 (88%)	

Question concerning the importance of the information regarding the management of tooth avulsion and dental first aid 255 (85%) of the school teachers considered this information important and remaining did not.

## DISCUSSION

The results of the study showed insufficient knowledge regarding tooth avulsion and its first aid treatment among primary school teachers of Davangere city, these results were comparable with previous similar studies.^5-8^ In present study, more than half of teachers did not know what was knocked-out tooth or tooth replantation. This is very surprising, since tooth avulsion occurs commonly in school children between 7 and 11 years old.^9^ However, the teachers themselves cannot be blamed for, since hardly any campaigning or exposure regarding tooth avulsion had been done in Davangere. In contrast, in a study conducted by C Blakytny et al in city of Cardiff at least one third of the teachers had received information dealing with avulsed tooth from campaign launched in 1989, where their community dental services had distributed a poster containing guidelines to be followed immediately after tooth avulsion.^10^

It is recommended by various studies that successful prognosis for avulsed tooth is immediate replantation with minimal further damage to cells of the root surfaces. In this study, 72 (24%) teachers had the knowledge of replacement of knocked-out tooth back to the socket while the others 228 (76%) did not. This might be due to unawareness of the teachers regarding replantation of avulsed tooth. However, in a study conducted by Hamilton et al only 10.7% of the respondents knew that the knocked-out tooth can be replaced back into its socket but they feared being sued for replanting the tooth incorrectly.^10^

Time is one of the important factor for avulsed tooth to preserve their vitality after replantation. Danish study had reported that teeth replanted within 5 minutes had the best prognosis. However, other studies suggest that 20 to 30 minutes is the maximum limit.^9^ In the present study, only 55 of the teachers answered that the tooth be replanted within 30 minutes. This result could be attributed to lack of knowledge and information regarding management of tooth avulsion.

In most of tooth avulsion cases, the avulsed tooth would fall on the ground and get dirty. The knowledge to clean a dirty avulsed tooth is also very important. In the present study, 43% teachers responded that they would clean the tooth in saline water, milk or tap water. However, 6% of them reported that they will brush the tooth root and crown to ensure the cleanliness of the tooth. Similar response was obtained in a study conducted by Hamilton et al, where 2.2% respondents wanted to scrub the tooth prior tooth replantation while only 8% washing it with milk.^10^ According to Raphael and Gregory, in their study they found that 15% respondents would scrub a tooth that was dirty before replanting it, unaware that they would severely decrease the chance of successful replantation.

On review of literature, the appropriate storage media to permit periodontal and pulpal healing are milk, saline water and saliva.^9^ However, most of time, saliva is the only quick media that is available. In present study, only 12% teachers had the knowledge to store the avulsed tooth in appropriate media such as milk and saline water. A total of 39% responded that they will put the avulsed tooth in cotton rolls as the avulsed tooth obviously will get contaminated with blood. In contrast, in another study teachers of Porto Rico statistically had more correct answer for transportation media for avulsed tooth.^9^ In a study by Raphael and Gregory, only 5% respondents knew that ‘milk’ was medium of choice for both washing and transporting avulsed tooth. The concepts of ‘dry storage’ among rural parents also indicate that there is lack of knowledge in this group on how avulsed tooth should be handled after accident. Studies show that dry storage during transportation would seriously prejudice normal healing and repair following replantation. However, some studies have demonstrated that even among dentists there has not been consensus on how the avulsed tooth should be stored.^11,12^ Westphalen et al in their studies found that dentists were well informed about tooth avulsion management.^13^

Regarding procedures to be followed in case of tooth avulsion, most teachers would take the child to the dentist nearby. Some studies have reported 50 to 60% of teachers would seek an emergency service nearby.^7,8,14,15^

A vast majority (85%) of teachers showed keen interest in knowing about the emergency management of knocked-out tooth compared with few others who did not. This may be because the teachers had good educational background, hence they were more enthusiastic in receiving knowledge about the emergency management of knocked-out tooth and they also had experienced such situations in their schools as the children spend most of the time in schools.

## CONCLUSIONS AND RECOMMENDATIONS

Based on the information collected from this study, it can be concluded that, there is lack of knowledge in the management of avulsed tooth in school teachers of Davangere city. They donot feel capable of immediate management of avulsed tooth by themselves.

As one of the child supervisors, all the school teachers should have the basic knowledge to recognize and assess the oral health problems of school children. Educational programs would be necessary to improve their awareness regarding the causative factors, prevention and the conservation of avulsed tooth to prevent its consequences in child’s future.
